# A founder mutation in the *PEX6* gene is responsible for increased incidence of Zellweger syndrome in a French Canadian population

**DOI:** 10.1186/1471-2350-13-72

**Published:** 2012-08-15

**Authors:** Sebastien Levesque, Charles Morin, Simon-Pierre Guay, Josee Villeneuve, Pascale Marquis, Wing Yan Yik, Sarn Jiralerspong, Luigi Bouchard, Steven Steinberg, Joseph G Hacia, Ken Dewar, Nancy E Braverman

**Affiliations:** 1 Department of Pediatrics, Université de Sherbrooke, Sherbrooke, Canada; 2 Department of Human Genetics, McGill University, Montreal, Canada; 3 Department of Pediatrics, Chicoutimi Hospital, Saguenay, Canada; 4 Department of Biochemistry, Université de Sherbrooke, Sherbrooke, Canada; 5 ECOGENE-21 and Lipid Clinic, Chicoutimi Hospital, Saguenay, Canada; 6 McGill University and Genome Quebec Innovation Center, Montreal, Canada; 7 Department of Biochemistry and Molecular Biology, Keck School of Medicine, University of Southern California, Los Angeles, USA; 8 Department of Neurogenetics, Kennedy-Krieger Institute, Baltimore, USA; 9 Department of Pediatrics, Centre Hospitalier Universitaire de Sherbrooke, 3001, 12e Avenue Nord, Sherbrooke, QC, J1H 5N4, Canada

**Keywords:** Zellweger syndrome, Founder effect, Peroxisome biogenesis disorders, Next generation sequencing

## Abstract

**Background:**

Zellweger syndrome (ZS) is a peroxisome biogenesis disorder due to mutations in any one of 13 *PEX* genes. Increased incidence of ZS has been suspected in French-Canadians of the Saguenay-Lac-St-Jean region (SLSJ) of Quebec, but this remains unsolved.

**Methods:**

We identified 5 ZS patients from SLSJ diagnosed by peroxisome dysfunction between 1990–2010 and sequenced all coding exons of known *PEX* genes in one patient using Next Generation Sequencing (NGS) for diagnostic confirmation.

**Results:**

A homozygous mutation (c.802_815del, p.[Val207_Gln294del, Val76_Gln294del]) in *PEX6* was identified and then shown in 4 other patients. Parental heterozygosity was confirmed in all. Incidence of ZS was estimated to 1 in 12,191 live births, with a carrier frequency of 1 in 55. In addition, we present data suggesting that this mutation abolishes a SF2/ASF splice enhancer binding site, resulting in the use of two alternative cryptic donor splice sites and predicted to encode an internally deleted in-frame protein.

**Conclusion:**

We report increased incidence of ZS in French-Canadians of SLSJ caused by a *PEX6* founder mutation. To our knowledge, this is the highest reported incidence of ZS worldwide. These findings have implications for carrier screening and support the utility of NGS for molecular confirmation of peroxisomal disorders.

## Background

Peroxisome biogenesis disorders (PBD; MIM #601539) are autosomal recessive disorders characterized by defective peroxisome assembly, division and/or impaired importation of matrix enzymes [1]. This results in multiple peroxisomal enzymes deficiencies, leading to developmental defects and progressive neurological involvement. PBDs are classified into two diagnostic groups, Rhizomelic Chondrodysplasia Punctata Type 1 (MIM #215100) and Zellweger spectrum disorder (ZSD), which includes the phenotypes of Zellweger syndrome (ZS) (MIM #214100), neonatal adrenoleukodystrophy (MIM #202370) and infantile Refsum disease (MIM #266510). ZS patients represent the most severe end of ZSD and present in the neonatal period with dysmorphic features, central hypotonia, seizures and multi-organ dysfunction. ZS is panethnic with an overall incidence estimated to be 1 in 50,000 births in the United States [2]. It is genetically heterogeneous and can be caused by mutations in any of 13 *PEX* genes [1,3]. Nevertheless, *PEX1* and *PEX6* mutations account for 70% and 10-16% of all cases, respectively [4,5]. In general, neither the clinical presentation nor specialized biochemical assays are predictive of the affected *PEX* gene [6]. Thus currently, hierarchal sequencing of a subset of *PEX* genes and exons, or complementation studies are used for mutation identification [4,5,7].

For decades, an increased incidence of ZS has been suspected in the Saguenay-Lac-St-Jean region (SLSJ) [8]. However, the incidence and molecular basis of these cases remained unresolved. The SLSJ region is located in the northern-eastern part of the province of Quebec, Canada and the vast majority of its population is of French-Canadian descent. Population genetics of SLSJ have been extensively studied and were marked by successive population bottlenecks, which contributed to shape its unique genetic pool [8,9]. By the English conquest (1608–1759), approximately 8500 French settlers had settled in Nouvelle-France [10]. A large number were from Atlantic seaports or around Paris but interestingly, a sizable fraction (about 25%) of individuals that settled in Côte-de-Beaupré (near current Quebec City) were from the old province of Perche, located in the south of present Normandy [11]. Pioneers migrated from Côte-de-Beaupré to Charlevoix around 1675 and then from Charlevoix to SLSJ around 1830–1850, creating two additional population bottlenecks [9]. SLSJ has remained relatively geographically isolated and has a current population size of approximately 285,000 individuals [9]. As a consequence of this history, founder effects have been described for several genetic disorders including tyrosinemia type I (MIM# 276700) [12,13], autosomal recessive spastic ataxia of Charlevoix-Saguenay (MIM# 270550) [14,15], sensorimotor polyneuropathy with or without agenesis of corpus callosum (MIM# 218000) [16,17] and Leigh syndrome, French Canadian type (MIM# 220111) [18]. Each of these genetic conditions shows a low genetic complexity in French-Canadians of SLSJ, with one or two mutations contributing to the disease frequency.

Given the population history of SLSJ and suspected increased incidence of ZS, a low allelic complexity would be expected. We reviewed ZS patients diagnosed in SLSJ over the past 20 years and investigated the molecular basis of these cases to identify the putative founder mutation amongst the possible *PEX* genes.

## Methods

### Patients and biological material

Patient records were provided by the genetics service of Saguenay (province of Quebec, Canada). Plasma very long chain fatty acids (VLCFA) were performed by gas chromatography–mass spectrometry [19] at the reference center for Quebec (Centre Hospitalier Universitaire de Sherbrooke). The parents of identified patients were re-contacted by the clinical service and written informed consent was obtained according to institutional guidelines. The study was performed with the approval of institutional ethics committee (McGill University Health Center). Genomic DNA was extracted from skin fibroblast cultures or whole blood of affected patients and parents (PureGene DNA purification kit, Qiagen).

### *PEX* gene sequencing

A total of 152 amplicons were designed to cover all exons and splice site junctions (~59 Kb) of the *PEX* genes implicated in ZSD (*PEX1*, 2, 3, 5, 6, 10, 11β, 12, 13, 14, 16, 19, 26) in addition to *PEX5L, PEX11 α* and *γ*. Primer pairs were chosen to obtain amplicon length of 350–400 bps and were tailed with GS FLX primers A and B sequences (Roche/454) (Additional file 1: Table A). Singleplex PCR was performed using the following condition (Additional file 1 for full details, Table A): 1X PCR buffer with 1.5 mM MgCl_2_ (Platinum® Taq, Invitrogen), dNTPs 200 μM each, forward and reverse primers 0.5 μM each (IDT, USA), DMSO 0% or 5%, DNA polymerase 0.025U/uL (Platinum® Taq, Invitrogen), 50 ng of DNA in a final volume of 20uL. Thermocylcler conditions were: initial denaturation 95°C x 5 min, then 35 cycles of 95°C × 30 seconds, 54°C or 62°C × 30 seconds, 72°C × 60 seconds, and final extension 72°C × 10 minutes. NGS was performed on pooled amplicons ( Additional file 1: Figure A). Amplicons were quantified on a Bioanalyser 2100 (Agilent technologies), pooled in equimolar quantity (50 ng each), and then purified on magnetic beads using Agencourt AMPure XP system. Barcode tailed with Titanium A and Titanium B sequencing primers (Roche/454) were then added to amplicons by PCR using the GS FLX primers A/B sequences, with the following conditions: 1X PCR buffer with 1.5 mM MgCl_2_ (HotStar Taq®, Qiagen), dNTPs 200 μM each, forward and reverse primers 0.8 μM each, DNA polymerase 0.02U/uL (HotStar Taq, Qiagen), 10 ng of purified pooled amplicons in a final volume of 25uL. Thermocycler conditions were as follows: initial denaturation 96°C for 15 minutes, then 10 cycles of 96°C for 45 seconds, 65°C for 30 seconds, 72°C for 60 seconds, and final extension 72°C for 10 minutes. Purification on magnetic beads using Agencourt AMPure XP was then performed and an aliquot was analyzed on the Bioanalyser to verify absence of significant primer dimers. Library preparation was performed as per manufacturer instruction and products were sequenced bidirectionally using the Roche/454 GS FLX Titanium platform. SNP/indel discovery was performed with NextGENe software, using an allele frequency cut off of 15% and a minimum of three reads for positive calls. A minimum Polyphen score of 20 was used to filter variations. Bidirectional Sanger sequencing was performed in parallel using ABI 3730XL DNA Analyzer according to manufacturer instructions. All data was reviewed with CodonCode Aligner v3.7.1 (CodonCode Corporation) for sequence variation. Mutations in *PEX6* are reported based on RefSeq NM_000287.3.

### Transcript analysis

Total RNA was extracted using RNAzol B (Cinna/Biotecx, Friendswood TX). For Northern analysis, 10 μg was applied per lane and electrophoresed at 40 V in formaldehyde gel overnight. Hybridization and washing were done as described [20]. For RT, we used SuperScript^TM^ II (Life Technologies), 10 pmol of poly dT primer and 5 μg of total RNA. PCR reactions contained 5 μl of first strand cDNA, 10 pmol primers, 100 μM dNTPs, 2.5 U TAQ polymerase and 1X buffer; cycling was 95°C x 6 minutes, 35 cycles of 56°C, 72°C and 95°C × 1 min each, 72°C × 10 min. cDNA was amplified from exons 1–3; PCR products were cloned and sequenced.

### Immunoblotting

Total cell lysates were collected as described [21]. 10 mg protein/lane was loaded on 7.5–12.5% SDS/ PAGE minigels and run at 170 V x 1 hour. Separated proteins were transferred to nitrocellulose membrane at 110 V and 4°C. Membranes were blocked, hybridized with PEX6 polyclonal antiserum (derived from amino acids 1–421, gift from Gabrielle Dodt, Universitat Tübingen, Germany) and visualized by ECL [21].

### Immunohistochemistry

Primary fibroblast cultures from control and PEX6 deficient patients, passage numbers 5–15, were cultured in Eagle’s minimal essential media supplemented with non-essential amino acids and 10% fetal calf serum at 5% CO_2_. Indirect immunofluorescence was performed as previously described [22,23]. Rabbit anti-thiolase and PMP70 were described [21]. Cells were visualized with an Olympus BX51 microscope at 60X oil immersion and digital images were taken. FITC and Texas Red labeled secondary antisera were purchased (Jackson ImmunoResearch Lab).

## Results

### ZS patients identified

The genetics service of Saguenay (province of Quebec, Canada) began its activity in 1988. Between 1990 and 2010, a total of 5 cases of ZS from four unrelated families were diagnosed based on clinical and either biochemical or histological data (Table 1). We reviewed all values of plasma VLCFA performed by gas chromatography–mass spectrometry [19] at the reference center for Quebec (Centre Hospitalier Universitaire de Sherbrooke), starting in 1992. Using a lower limit of 2 μM for C26:0, we identified 14 potential ZSD cases in the province of Quebec during the period 1992–2010. Two of them were known patients of the genetics service of Saguenay (patients C1 and D1; Table 1). Of the remaining 12 potential cases, none come from the SLSJ region and they were therefore excluded from this study. Inspection of three-generation pedigrees in these 5 cases did not identify any close relationship between the families, aside from two cases in the same sibship. Clinical data from medical charts were reviewed by two medical geneticists with expertise in peroxisome disorders (NEB and SL) for clinical presentation, autopsy report, skeletal survey and specialized biochemical genetics laboratory analyses (Table 1). Cases were included for molecular analysis when there was evidence of at least one defective peroxisomal pathway or absent/abnormally shaped peroxisomes on liver biopsy. All cases presented in the neonatal period and affected individuals died within the first year of life.

**Table 1:** Zellweger syndrome patients diagnosed in Saguenay-Lac-St-Jean

**Family- patient#**	**Age of death (months)**	**Clinical presentation**	**VLCFA C26:0**^**1**^	**VLCFA C26 / C22**^**2**^	**PLG synthesis**^**3**^	**Liver biopsy**
**A1**^**4**^	0.5	Hypotonia, seizures, brain malformations (hypoplastic corpus callosum, nodular heterotopia, dysplastic dentate nucleus), large fontanelle, clouded cornea, ASD, bilateral microcystic kidneys, elevated liver enzymes, severe jaundice, hepatomegaly with microvesicular steatosis on autopsy, ectopic patellar calcifications.	ND	ND	ND	Absence of peroxisomes
**B1**^**4**^	<12	Hypotonia, brain malformations (agenesis of corpus callosum, ventriculomegaly), kidney disease (?glomerulosclerosis), cryptorchidism, ectopic patellar calcifications, bilateral club feet.	2912 μg/ml (plasma)	0.61	ND	ND
**C1**	5	Prematurity and intrauterine growth retardation, hypotonia, brain malformation (polymicrogyria), large fontanelle, increased nuchal fold, VSD, renal cysts, ectopic patellar and cervical calcifications, agenesis of cervical vertebrae (C3).	3.37 μM (plasma)	0.68	ND	ND
**D1**	6	Meconial amniotic fluid, respiratory insufficiency, hypotonia, seizures, nystagmus, deafness, brain malformation (ventriculomegaly), large fontanelle, increased nuchal fold, hepatosplenomegaly, equinovarus deformity, cryptorchidism, ectopic patellar calcifications, low platelets.	22.53 μM (plasma)	0.63	ND	ND
**D2**	0.5	Meconial amniotic fluid, respiratory distress, seizures, brain malformations (ventriculomegaly), large fontanelle, congenital cataracts, jaundice, ectopic patellar calcification.	0.79 μg/mg (CVS)	2.86 (CVS)	4.43 (CVS)	ND

VLCFA: very long chain fatty acid; PLG: plasmalogen; CVS: chorionic villous sample; VSD: ventricular septal defect; ASD: atrial septal defect; ND: not done

^1^Normal values: plasma 0.33 ±0.18 μg/mL (mean ± SD) or 0.11 – 0.89 μM (5^th^-95^th^ centile), CVS 0.05 ±0.02 μg/mg (mean ± SD); ^2^Normal values: plasma 0 – 0.030 (5^th^-95^th^ centile), CVS 0.10 ±0.04 (mean ± SD), ^3^ Ratio of ^3^ H (ER steps in plasmalogen synthesis) and ^14^C (peroxisomal steps in plasmalogen synthesis)): normal 0.67 ± 0.19 (mean ± SD) and ZSD 9.92 ± 4.4. ^4^A1 and B1 were diagnosed prior the establishment of the VLCFA reference laboratory in Quebec.

### Next generation sequencing

**Figure 1:**
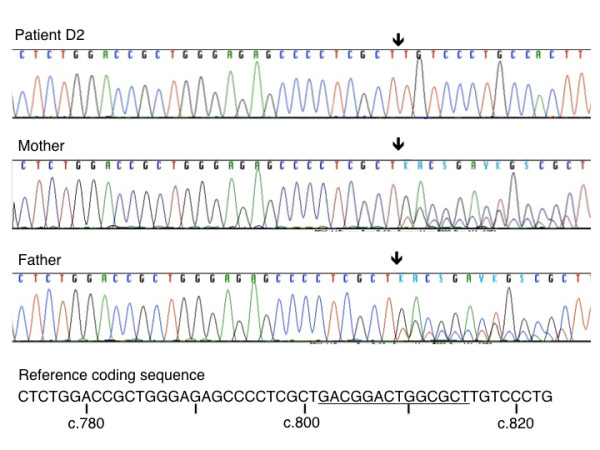
*** PEX6 *founder mutation c.802_815del.** Sanger sequencing traces are shown for patient D2 (homozygote), and his mother and father (heterozygotes). The reference sequence is shown at the bottom with the deleted sequence underlined (Ref Seq NM_000287.3). The arrow indicates the position of the 14-bp deletion. *PEX6* exon 1 extends from nucleotides 1–882

Patient D2, who had defective VLCFA oxidation and deficient plasmalogen synthesis was selected for sequencing of all known human *PEX* genes on our NGS platform. A total of 113,209 reads of an average length of 303-nt were obtained. 73% of reads mapped to the target sequences, with an average coverage depth of 452 reads / base. More than 99% of targeted exons were covered by at least one read and 94% of target exons ± 50 bp were covered. Sanger sequencing was performed in parallel on this sample. Coding sequences and flanking 50-bp intronic sequences were analyzed for sequence variations. Ten variations with a Polyphen score of 20 or more were identified using NGS among the *PEX* genes, 9 of which were located in intronic regions. A homozygous mutation was found in *PEX6* exon 1 (c.802_815del) and confirmed by Sanger sequencing (Figure 1). DNA samples of four other SLSJ patients diagnosed with ZS (Table 1), or if unavailable, DNA from parental obligate carriers was obtained and *PEX6* exon 1 was subjected to Sanger sequencing. All four cases harbored the same homozygous *PEX6* mutation with parental heterozygosity confirmed in all cases.

### Functional analysis of the identified *PEX6* mutation

**Figure 2:**
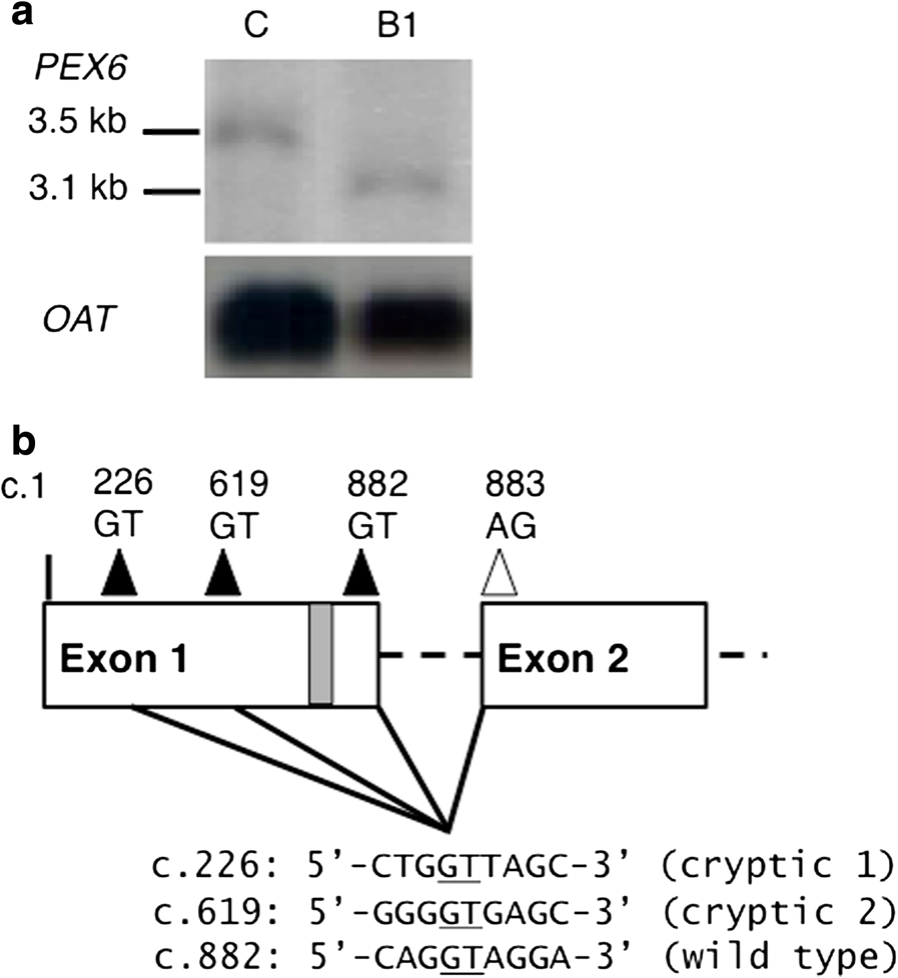
** Use of alternative cryptic donor splice sites in *PEX6 *c.802_815del mutant cells.** (**a**) Northern blot analysis of fibroblast total RNA of patient B1 compared to control, and (**b**), graphical representation of *PEX6* exon 1 and 2 showing the normal 5’-splice donor (GT) and 3’-splice acceptor (AG) sites, and the two upstream cryptic splice donor sites utilized. Sequences flanking the cryptic and wild type splice donor sites are provided. The grey box represents the c.802_815 deletion

Fibroblasts from patient B1 were available for further study of the cellular consequences of the identified mutation on *PEX6* transcript and protein. To evaluate the transcript produced from this allele, we performed Northern blot analysis of total cellular RNA, revealing a shortened * PEX6 * transcript, estimated to be around 200–600 nt shorter within the resolution of the northern blot (Figure 2a). RT-PCR, followed by sequencing of cDNA clones obtained showed two different internally deleted, but in-frame transcripts, corresponding to the loss of the terminal sequence of exon 1 (c.226 to 882 and c.619 to 882; Figure 2b; see Additional file 2: Figure B for full sequence of the alternate transcripts).

**Figure 3:**
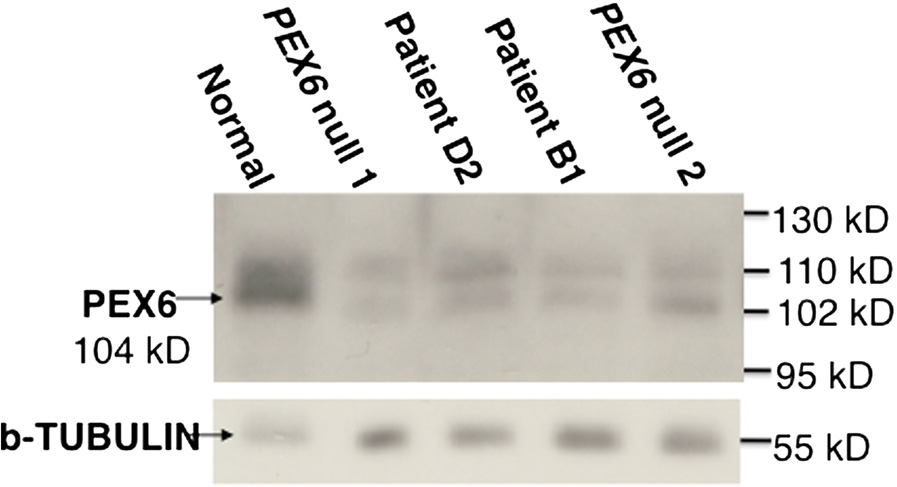
** PEX6 protein levels evaluated by immunoblotting.** Results from cultured fibroblasts obtained from ZS patients B1 and D2 (both homozygous for *PEX6 * c.802_815del) are shown along with two PEX6 null mutants, and a normal control. Genotypes for *PEX6* null mutant 1: c.[499_500del]; [2095-10_21del] and *PEX6* null mutant 2: c.[1314_1320del];[2472-2A > G]. The results from b-tubulin analysis are provided as loading control

To determine whether an internally deleted PEX6 protein is produced, we performed immunoblotting on whole cell lysates using a PEX6 polyclonal antibody (Figure 3). Similar to two other PBD patient fibroblasts with *PEX6* null mutations, the *PEX6* c.802_815del homozygote mutant fibroblasts did not produce detectable amounts of either full-length or truncated PEX6 protein. We note that a non-specific upper band (~110 kD) is observed in all samples and that all *PEX6* mutants, irrespective of genotype, show a lower band around 102-kD, not seen in the control sample. Since the two *PEX6* null cell lines evaluated had no detectable *PEX6* RNA on northern blot analysis (data not shown), we conclude that PEX6 protein is not produced in any of these cell lines.

**Figure 4:**
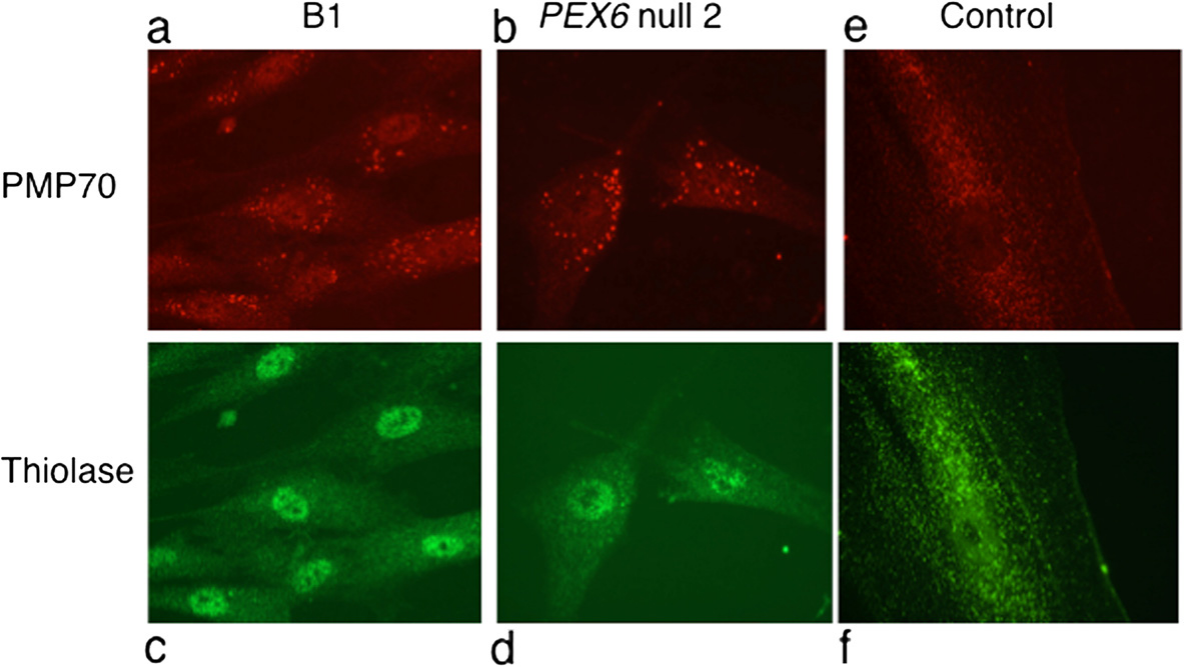
** Decreased peroxisome number and matrix protein import in cultured fibroblasts.** Immunofluorescence-based peroxisome assembly assays were performed on cultured skin fibroblasts obtained from ZS patient B1 and the same PEX6 null cell line used in Figure 3 (Panels a-d) and healthy control (Panels e and f). Briefly, cells were fixed, permeabilized and stained with anti-human PMP70 (secondary, texas red) and thiolase (secondary FITC) antiserum to highlight peroxisome membrane and matrix proteins, respectively

The absence of residual PEX6 protein should correlate with reduced numbers of enlarged peroxisomes and negligible matrix protein import observed in fibroblasts from Zellweger syndrome patients. Thus we performed indirect immunofluorescence on fibroblasts from patient B1, the *PEX6* null cell line 2 and a control, using the peroxisomal membrane marker, PMP70, and matrix protein marker, thiolase (Figure 4). We found a severe decrease in peroxisome number, abnormally enlarged peroxisomes and absent matrix protein import into the organelle in the patient, compared to control fibroblasts. These results are consistent with previous reports in Zellweger syndrome cells [24]. The similarity of the results between B1 and the *PEX6* null cell line 2 provides additional support for the lack of function of the *PEX6* c.802_815del allele.

### Incidence of ZS in SLSJ

Based on the five molecularly confirmed cases, we estimated the incidence of ZS in SLSJ. During the study period, a total of 60,954 live births were recorded in SLSJ ( http://www.stat.gouv.qc.ca/), which yield to 1 case per 12,191 live births (95% CI interval: 1/5,224 – 1/37,544). Under the assumption of Hardy-Weinberg equilibrium, the population carrier frequency in SLSJ is estimated to 1 in 55.

## Discussion

Founder effects have been described in SLSJ for several genetic disorders [12-18], which is not unexpected given its unique population history [9]. Here, we report a single *PEX6* mutation in all ZS cases in French-Canadians from the SLSJ region. There is strong evidence that this is a founder mutation given the elevated incidence of ZS in the population and no *PEX6* mutation hotspots have been identified large patient cohorts [25]. In addition, three-generation pedigrees of the four affected families from SLSJ did not reveal any close relationship that could otherwise explain the observed homozygosity for this mutation.

*PEX6* is the second most frequent gene involved with 10-16% of all ZS cases. In more than 100 ZS patients with *PEX6* mutations, c.802_815del was observed only 3 times in other populations, in patients with compound heterozygosity for this allele and a second, different *PEX6* allele. One was a US patient with unknown ethnicity [26] (also listed as http://www.dbpex.org, PEX6_30001), the second was of French-Canadian descent but not clearly from the SLSJ region ( http://www.dbpex.org, PEX6_00039), and the third was from a European population [25]. In cells from the US patient, Matsumoto and colleagues showed the presence of a shorter *PEX6* transcript due to the loss of 264 nucleotides at the end of exon 1 by RT-PCR. We confirmed the presence of a shorter *PEX6* transcript by Northern blot analysis in cultured fibroblasts from patient B1. However, the sequencing of RT-PCR products showed two different transcripts corresponding to the loss of terminal sequence of exon 1, c.226_882 and c.619_882. Therefore, the mutation should be described as (c.802_815del, p.[Val207_Gln294del, Val76_Gln294del]). Splice site prediction (NetGene2, v2.4) [27] performed on exon 1 revealed the main donor splice site at position c.882 as well as two additional cryptic splice sites at c.226 and c.619. Additional cryptic splice sites in large exons are not unusual [28]. Based on the ESEfinder algorithm [29,30], the SF2/ASF exonic splicing enhancer is predicted to bind the c.800-806 5’-CTGACGG-3’ sequence. Therefore, the c.802_815 deletion would destroy the SF2/ASF binding site needed for donor splice site definition at c.882, favoring the use of alternative cryptic splice sites. The predicted in-frame deleted PEX6 proteins are likely to be unstable and degraded given the classical Zellweger phenotype of these patients and negligible PEX6 protein found by immunoblotting. The 102-kDa band observed is unlikely to represent the product of the shortened transcript seen in c.802_815del mutants given that it was observed in two different *PEX6* null mutants not containing this allele. Moreover, the predicted in-frame loss of 88 amino acids (c.619 to 882) would lead to a protein of approximately 95-kDa or even smaller if the second predicted cryptic splice site is used (c.226), and this was not observed on the immunoblots.

Based on molecularly confirmed ZS cases over a 20 year-period, we estimated the incidence of ZS in SLSJ region to 1 / 12,191. To our knowledge, this is the highest incidence worldwide, although our confidence interval remains large (1/5,224 – 1/37,544). Since there is a single genetics service in SLSJ region, it is unlikely that recognized cases were missed. Moreover, no additional cases were identified through the Quebec reference center for analysis of very long chain fatty acids in blood samples. Compared to SLSJ, incidence of ZS has been estimated to 1 / 50,000 in the United States based on data from the reference center, the Kennedy-Krieger Institute, Baltimore, Maryland [2]. Data from the PBD reference center in Japan, showed a much lower incidence with 24 cases over a 10-year period (1 / 500,000); however, the incidence in the Okinawa Islands, 1/30,000, appears much higher [31]. An increased incidence of ZS was also proposed in a small Arab community (Karaite) inside Israel [32].

The increased incidence of ZS and the identification of a single *PEX6* founder mutation in French-Canadians of SLSJ raise the possibility of population-based carrier screening. The lethality of the condition, availability of prenatal diagnosis, and a technologic platform and infrastructure for population screening in SLSJ, argue in favour of carrier screening. Currently, voluntary population carrier screening is offered in SLSJ for four autosomal recessive conditions with increased incidence (tyrosinemia type I, autosomal recessive spastic ataxia of Charlevoix-Saguenay, sensorimotor polyneuropathy with or without agenesis of corpus callosum and Leigh syndrome French Canadian type). If we consider ZS, with an *a priori* estimated carrier frequency of 1/55, about 3,000 individuals will have to be screened to find one couple at 25% risk of having an affected child. This is about the number of live births per year in SLSJ ( http://www.stat.gouv.qc.ca/). However, larger scale studies that more accurately assess *PEX6* mutation carrier frequency in French-Canadians of SLSJ are needed prior to discussing the implementation of *PEX6* mutation screening.

Identification of mutations in ZSD samples is currently done using hierarchal Sanger sequencing of a subset of *PEX* gene exons or cell complementation assays followed by sequencing of the identified mutant *PEX* gene [4,5]. Both methods are time consuming and associated costs limit test accessibility, particularly for Sanger sequencing. NGS technologies enable the simultaneous analysis of hundreds of genes from multiple individuals at a favourable cost per test [33,34]. In our present study, we used targeted NGS to screen all known *PEX* genes for sequence variants and identify the candidate founder mutation. Validation of our *PEX* gene panel showed that mutations could be missed when coverage was lower than 20 reads per base [35]. Consequently, deep sequencing (>400X) was obtained to ensure that all targeted sequences were covered and to minimize risk of false negative calls considering variability of read numbers across targeted sequences. Given the size of this gene panel (<100-Kb), the protocol could be adapted to personal next generation sequencers, which require modest infrastructure investment [34]. The rapid and cost-effective nature of targeted NGS makes it an attractive alternative to current *PEX* gene mutation detection methods and may afford greater sensitivity in cases where biochemical diagnosis is illusive. For example, Ebberink and colleagues reported variations in Pex11β causing a ZSD phenotype in a patient who failed to show consistent plasma biochemical abnormalities indicative of global peroxisome dysfunction [3].

## Conclusion

In summary, we report increased incidence of ZS in French-Canadians of SLSJ caused by a *PEX6* founder mutation. To our knowledge, this is the highest reported incidence of ZS worldwide. These findings have implications for carrier screening and support the utility of NGS for molecular confirmation of peroxisomal disorders.

## Abbreviations

NGS, Next generation sequencing; PBD, Peroxisome biogenesis disorders; SLSJ, Saguenay-Lac-St-Jean; VLCFA, Very long chain fatty acids; ZS, Zellweger syndrome; ZSD, Zellweger spectrum disorders.

## Competing interests

The authors declare that they have no competing interests.

## Authors’ contribution

SL participated in the study design, optimization of amplicons, amplification of *PEX* genes panel, analysis of NGS reads and clinical data, and drafted the manuscript. CM and JV participated in the recruitment of patients and acquisition of clinical data. JV also participated in the drafting of the manuscript. SPG and LB performed the analysis of Sanger sequencing traces of the *PEX* genes panel. SS performed *PEX6* Sanger sequencing confirmation for patients and parents. PM participated to the analysis of NGS reads. KD participated in study design and in the analysis of NGS reads. SJ carried out the immunoblotting assay. WYY performed the primers design and participated in optimisation of amplicons amplifications. JGH participated to the study design and drafted the manuscript. NEB participated in the study design and clinical data, analysed immunohistochemistry and northern experiments, and drafted the manuscript. All authors were involved in revising the manuscript. All authors read and approved the final manuscript.

## Pre-publication history

The pre-publication history for this paper can be accessed here:

http://www.biomedcentral.com/1471-2350/13/72/prepub

## Supplementary Material

Addtional file 1 Word document, Supplementary methods including primers sequences for targeted amplification of PEX genes and PCR conditions (Table A) and next generation sequencing workflow (Figure A). Click here for file

Addtional file 2 Word document, cDNA sequences and predicted proteins of alternate transcripts (Figure B). Click here for file
