# Supplementary material for: A founder mutation in the PEX6 gene is responsible for increased incidence of Zellweger syndrome in a French Canadian population
Source: BMC Med Genet. 2012 Aug 15;13:72. doi: 10.1186/1471-2350-13-72 (PMC3483250; doi:10.1186/1471-2350-13-72)
Supplement: Addtional file 1 — Word document, Supplementary methods including primers sequences for targeted amplification of PEX genes and PCR conditions (Table A) and next generation sequencing workflow (Figure A). [file 1471-2350-13-72-S1.doc]

**Supplementary methods**

**Table A. Primers sequences for targeted amplification of PEX genes**

| **Gene** | **Exon(s)/**  **amplicon** | **Forward primer (5’ – 3’)** | **Reverse primer (5’ – 3’)** | **Annealing (°C)** | **DMSO**  **(0% or 5%)** |
| --- | --- | --- | --- | --- | --- |
| *PEX1* | 1 | actacaggcttacggcagga | actggacttgtgggatccag | 62 | 5 |
|  | 2 | catgtgaatccagaaaaatg | ccaaaattaggggatgtgga | 54 | 0 |
|  | 3 | acctaaaatggtaagaagat | ggactaatggcatgcactacc | 54 | 0 |
|  | 4 | tgatcttttcgtctgttggttc | TCAGTGATTCCCACAGTATTTGA | 54 | 0 |
|  | 5a | GGAAGAGACCAGAAAGGAATGA | TTTGCTGTTGCTTTGGAGAA | 62 | 0 |
|  | 5b | ATAACGCGTCAGCAACCTCT | tggggatgtttaagccacat | 54 | 0 |
|  | 6 | cctgcatggactgagacaac | aaatggcgaacatggcatat | 54 | 0 |
|  | 7 | cagacctgtgcctatagaac | gctgggattacaggcatgag | 62 | 0 |
|  | 8 | gcagcagagtgagaccctgt | ttgtgctaaggcattcattt | 54 | 0 |
|  | 9 | ccctggatgggtacattgtt | ggcgtgaagatagagacaatcc | 54 | 0 |
|  | 10 | ctgggaaggcaaaattcagc | cctatataatagatggtc | 54 | 0 |
|  | 11 | aggctgaatcttggtggttg | ttcagagtggtgtcagtgagc | 54 | 0 |
|  | 12 | cgagcttttctagtgctttc | gactaaaaatgctgactgac | 54 | 0 |
|  | 13 | ggtcaaccttagagtatcat | gcctctagcacaatatgcac | 54 | 0 |
|  | 14 | cactatagatttgtcaacctg | gaaagaagattccaagttcagg | 54 | 0 |
|  | 15 | ttccagctaagatgatggca | cagagtgaggtctcaattctaa | 54 | 0 |
|  | 16 | tggtcgtgattaccatctgaa | gcaagaggaggagtggaatg | 54 | 0 |
|  | 17 | gggcagaagcatcaagcata | gagaatccacgtcctctagc | 54 | 0 |
|  | 18-19 | ccaactatgaagcctgattc | acctggcagaagtaaagctc | 54 | 0 |
|  | 20 | aggctgttggtacatttctca | gaaattttgacattgtacttc | 54 | 0 |
|  | 21 | gctctttgaaaaatgcttct | tatgtggggctggttaggag | 54 | 0 |
|  | 22 | ttctctccccctcttccttc | aagcaaggaagcacataacca | 54 | 0 |
|  | 23 | tgcttccttgctttcaatgtt | ttctgacattttggggttttt | 54 | 0 |
|  | 24a | gtagtatttatacatattaa | TTAGTTCCACCATTTGGCTA | 54 | 0 |
|  |  |  |  |  |  |
| *PEX2* | 4a | tgaggaggttgtacaagattgc | CATAGCATCGTTCTTCTAAC | 54 | 0 |
|  | 4b | CTAACCTGAGATATCAGCCA | GTCACTATTAGGTGCACCAG | 62 | 0 |
|  | 4c | CAAGCTGTCTTCATGGTGTA | TTAGATTTCAGTCATGCCTG | 54 | 0 |
|  |  |  |  |  |  |
| *PEX6* | 1a | ctcctcgttggtgtcctgtc | CCAAGCAGTGCCCAACCTAG | 62 | 5 |
|  | 1b | CGGCTCCTGGCACTGGGCTC | CAAGGCCACGGAGACAGCTC | 62 | 5 |
|  | 1c | AGGGACTGGAGATTCACTAG | gaaagaggaagcactcctgac | 62 | 5 |
|  | 2 | gagaaggttataacgtggtg | gagggtgagaagctatcctc | 54 | 0 |
|  | 3 | aaagagggctccaaaagagc | tgacaacgaaatcctcttgg | 54 | 0 |
|  | 4-5 | gactctggatctggggttca | aactacccatctggctgctg | 62 | 0 |
|  | 6 | ggctgttggatatgctggat | gaagtgctagggcctgtgag | 62 | 0 |
|  | 7 | agtccagggaatgggatagg | tgagaggcccagctctgtat | 54 | 0 |
|  | 8 | tggggaacactcggttaaag | tcactcacaaggcaacagga | 54 | 0 |
|  | 9-10 | tgctcatgtgtcctttcctg | cgtggttgggatatgctctt | 54 | 0 |
|  | 11 | aagagcatatcccaaccacg | ctgatccacccaccatcc | 54 | 0 |
|  | 12 | gacggctctcaccttctcac | gggcaggaacctgacttgta | 54 | 0 |
|  | 13 | ctgtattgtcaggccccagt | cactcagctgtgcccaatgt | 62 | 0 |
|  | 14-15 | ctaagggggacaccaaggac | cagagttggcatcacctcct | 62 | 5 |
|  | 16 | aggaggtgatgccaactctg | ccccactagctttttggttg | 54 | 0 |
|  | 17a | ctgcctgggcatagaccaca | CTGTTGCTGCTGTCTCAATG | 62 | 0 |
|  |  |  |  |  |  |
| *PEX7* | 1 | cggaccaatcagaatctcaaa | cactcggcaaagaaaggaga | 62 | 5 |
|  | 2 | ttaccctggcaggttcaaag | gaggggaagactgcactgag | 54 | 0 |
|  | 3 | aactcccgtagttgtgcagat | catccccaaatggatttcaa | 54 | 0 |
|  | 4 | tggaggaatggacaagatca | aaagctcagctgtcgaatca | 54 | 0 |
|  | 5 | tcagcattagtttggccctt | ctcctgttttctctctgaagc | 54 | 0 |
|  | 6 | tcaagtggtgtgatgggaaa | acataaggcagcttcccaaa | 54 | 0 |
|  | 7 | tcaaaaatgactccttggttca | cattgcccaaattcattcaa | 54 | 0 |
|  | 8 | gccagaagatttttgcctca | tggatcacgaaaacaggaaa | 54 | 0 |
|  | 9 | ggctgagcctgtttggataa | tcctgggaaggttccttttt | 54 | 0 |
|  | 10a | acgtttcttcttacttcatt | ATCAGGCTTAACGAGTCAGC | 54 | 0 |
|  |  |  |  |  |  |
| *PEX10* | 1 | tgcgcctccgactaccttcc | ctccatgaggggcaacac | 62 | 5 |
|  | 2 | gagtgagtgttggcttcatg | tccaggagcttctcacactg | 54 | 5 |
|  | 3 | cagtctcagggtccacatac | gccctcagcgcctgctactt | 62 | 5 |
|  | 4 | gtgccaactgaacgtgtgac | ccttacaggtccttgtgaag | 62 | 5 |
|  | 5 | cttcacaaggacctgtaagg | ctcaaaactggagggtgctc | 62 | 5 |
|  | 6a | tgcagagaatgtggacagga | GGAAGGGAGGGAAACCTAAA | 62 | 5 |
|  |  |  |  |  |  |
| *PEX12* | 1b | TACCCCCTCGCCACATACTA | ctaggctaccaaataagcac | 62 | 5 |
|  | 2a | ggtgtagaacttgtgtaatg | GGGAAGAAGGGGGATGAATA | 54 | 0 |
|  | 2b | GAGAAGCTGGTTTCTAGCCT | ccaggatgacagtccttgttt | 54 | 0 |
|  | 3a | tgcctccttgttcttttctcc | GTGAGGATAAGACATGATTC | 54 | 0 |
|  |  |  |  |  |  |
| *PEX26* | 2 | tgggcaaagagatgaggact | gcaatctgggaaggacagaa | 62 | 5 |
|  | 3 | ctgagttgggagctggagag | gatgaaaatcctctcgtttc | 54 | 0 |
|  | 4 | gtggtcatgggaaatgaacc | acagaccccgaggtacagtg | 54 | 0 |
|  | 5 | gtagagacggggtttcacca | gtaggactctgacaaacacc | 62 | 5 |
|  | 6a | gggtgggacgttcactgtag | GCCTTCACCAAGAAGATGGA | 54 | 0 |
|  |  |  |  |  |  |
|  |  |  |  |  |  |
| *PEX3* | 1 | gtttgtgatttcgggagagc | tggttgatcgaactccctct | 62 | 5 |
|  | 2 | gtgcccatcatttcctctgt | atttgatcccggtgatatgc | 54 | 0 |
|  | 3 | atgcgcatttcttagtgagg | gccttgaatgctttatgcaa | 54 | 0 |
|  | 4 | cgtattttgtgggcttgctt | aggatgtgctgtcacacagaat | 54 | 0 |
|  | 5 | aggatggttcatgattttac | tttgaacctcctgagggaaa | 54 | 0 |
|  | 6-7 | tttttcccgtcatttcagat | ccgtttgtcaagtcactggtt | 54 | 0 |
|  | 8 | ggcccttaactagtggctga | tggtttggttaaatggttatgaa | 54 | 0 |
|  | 9 | tgggggagggtctaactttt | tgcttccctccagtttgagt | 54 | 0 |
|  | 10 | tgttggtggctttcaaaggt | aaaaagacaagcaaagcacca | 54 | 0 |
|  | 11 | cgccaaaagaaattggtcat | gacaaacatgaccaccacca | 54 | 0 |
|  | 12a | agctatatgttttgcaaact | CAATTCTGATATTTGCACGT | 54 | 0 |
|  |  |  |  |  |  |
| *PEX13* | 1 | cgatgagcctttaccctgag | gggaagggaatacctccact | 62 | 5 |
|  | 2a | gaattgaatttattgtatgc | TAGCATCCATCATCATACTG | 54 | 0 |
|  | 2b | CAAGCTGAAGAAAGCAGCAG | tctgaacctgttcccttacagt | 54 | 0 |
|  | 3 | ggggaggacttttctcttgg | taactctgctggggcatttc | 54 | 0 |
|  | 4a | tgttggacctccaaagtata | ATGTTTTCATTGGATTGTAC | 54 | 0 |
|  |  |  |  |  |  |
| *PEX14* | 1 | acatcgacaggctctgttcc | gagactcggggtccctacc | 62 | 5 |
|  | 2 | tgggtggcactgtgatctta | tctggggtggatgcttttag | 62 | 5 |
|  | 3 | agccagtgaagacagggaaa | atgagtgggcagctctagga | 62 | 5 |
|  | 4 | aatcggggaggcaacttact | tcccagtttctcctggtttg | 54 | 0 |
|  | 5 | gagtgttccacttgcccatt | ccagtgaaagggtgctgaat | 54 | 0 |
|  | 6 | gcggcagaatttgagagatt | gctgtgaggggtgacttgat | 54 | 0 |
|  | 7 | cgtctgtgcctgtgaatttg | ccaagggacaagagtgaagg | 54 | 0 |
|  | 8 | ccaaacagagctgtgggaat | gagctcccagtcctcctttc | 62 | 5 |
|  | 9a | caacctcctccccttcttct | CTCCACCTGCTCGTTGATCT | 54 | 0 |
|  | 9b | CATGTGGACGAGGAGGACTG | AAGGCGTCTGTCCGGGAGAG | 62 | 5 |
|  |  |  |  |  |  |
| *PEX16* | 1 | gcggagagcgtgctaaccaa | cagggcccagaaggaactga | 62 | 5 |
|  | 2 | acccgaagacgaaatgtgag | tcagataaggtccccagacg | 62 | 5 |
|  | 3 | agcagagcatgtgacaggtg | atggtttgaggctcagatgg | 54 | 0 |
|  | 4-5 | acttcttggcctggggttct | agggcttggggaagtagctc | 62 | 5 |
|  | 6-7 | gccttctgtctatccctgct | agcacactccccactcaatc | 62 | 5 |
|  | 8-9 | cctgtgctggagacatcctt | tcagggtgtcctgggtgctt | 62 | 5 |
|  | 10 | gtgtaccgggctttgtccta | ccgacttcctctcctgactg | 62 | 5 |
|  | 11a | cccacctggcagaaaatcta | CAGCAGTGTTCGCTCCTATG | 54 | 0 |
|  |  |  |  |  |  |
| *PEX19* | 1 | ttccgcactggtcaacaata | tcacttaacccccagaatgg | 62 | 5 |
|  | 2 | agccttgcttgcattctcat | aaggtaccctcccacattcc | 54 | 0 |
|  | 3 | gtgcatttcccatagccact | tggtgttttcaggcaacaaa | 54 | 0 |
|  | 4 | tcctaaatgacgctggcttt | cagcaacagacttgggatga | 54 | 0 |
|  | 5 | agagtggtgagcatcccaag | caactctccctgcctcaaat | 54 | 0 |
|  | 6 | ccaggtgatctggttggagt | cacctaagtggacagcacca | 62 | 5 |
|  | 7 | ctgggcttttctctatttca | CAGACTTCTCCCGCAGGTGA | 54 | 0 |
|  |  |  |  |  |  |
| *PEX11a* | 1 | aagtccgcgaagttgctcta | aggaggggctcggatactaa | 62 | 5 |
|  | 2 | tgttgtctggaggcatgtgt | caaacctgttgtgccatgag | 54 | 0 |
|  | 3a | ttcactattgtgggttctac | GGAGGATGCTGCTTCAGAGA | 54 | 0 |
|  | 3b | AGCATCCCAGGATCCTCTTT | GTTCTTGGCCTCATCTCTGC | 54 | 0 |
|  |  |  |  |  |  |
| *PEX11b* | 1 | ggccaagctttcttttctga | cggagttgacccttcttgac | 62 | 5 |
|  | 2 | gaggaggaaggggagtgttc | ggaaaggttgggggataaaa | 54 | 0 |
|  | 3 | gcctctccacttacaaatcc | agctatgcaatatagaggct | 54 | 0 |
|  | 4a | tgcccttactgacccttttg | CTTGAGTCGTAGCCAGGGAT | 54 | 0 |
|  | 4b | GTCCTCCATCCTGTCTATTC | CTTACAGGCCAAACCTTATA | 54 | 0 |
|  |  |  |  |  |  |
| *PEX11g* | 1 | cccacctctgcaaactctgt | gggccaaactctaggacctc | 62 | 5 |
|  | 2 | gccacactgactgcttctg | tactccctagagccccacaa | 54 | 0 |
|  | 3 | gggcagtgggtaagtctg | gggccgctgccactttgc | 62 | 5 |
|  | 4 | ttgtggagctaaccctcctg | agcaagcctcagtttctcca | 62 | 5 |
|  | 5a | caggtgtgagcgtgagtgtaa | GTTTCACCACAGGCAGCTCC | 62 | 5 |
|  |  |  |  |  |  |
| *PEX5-isob* | 3 | gcttgggtacctcagttcca | aaaggtgcacagggttttct | 54 | 0 |
|  | 4 | agatgcctatgggcttcatc | CAGGAATTCAGCCACCAAct | 54 | 0 |
|  | 5 | caatttttgggctttcctga | tcccctcccactagactctg | 54 | 0 |
|  | 6 | cacgtggattcagggttctt | gccaaccaaactcttcgttc | 54 | 0 |
|  | 7 | ctccttgagaatgggttgga | gttggttcgagaacgtggat | 54 | 0 |
|  | 8 | taggagtggggagtagcatg | aggcaaggagatgagggaat | 54 | 0 |
|  | 9 | tgcagctagagatggtcagg | caccaacctgggtactt | 54 | 0 |
|  | 10 | ttccagtcccacttcagagc | gatgatccaggtccactatg | 54 | 0 |
|  | 11 | atcccagcagagctgagtgt | tgtgcaaacctccttcacag | 54 | 0 |
|  | 12 | gccacctctcagtccatctc |  | 54 | 0 |
|  | 13 |  | agcacctcacccacaccttt | 54 | 0 |
|  | 14 | gagtcacaggtgaggccatt | cctaggtttcccatcccatt | 54 | 0 |
|  | 15 | aatgggatgggaaacctagg | ttcccaacaaatggctcttc | 54 | 0 |
|  | 16 | ggaggactgggaaaagaagg | aaggcagggagaaaaagctc | 54 | 0 |
|  | 17 | aaccagccctagctttctcc | ccgtaccgcttatggtcatc | 54 | 0 |
|  |  |  |  |  |  |
| *PEX5R* | 1 | gaaagagttcggcgaagaag | cccccaagaattctctccag | 54 | 5 |
|  | 2 | tgggtagtggaggctaagca | ccagagaagggctgaacact | 54 | 0 |
|  | 3 | cttgcagcagcctacctctt | tagcaattttggttttcatc | 54 | 0 |
|  | 4 | gccctttgacacctctgttc | tgttctgcaaaattggttgg | 54 | 0 |
|  | 5 | atgcagcaaacatcccagtc | tgagcatccatagccgcaag | 54 | 0 |
|  | 6 | tcaatgaagctcccttggat | aatggccgttttctggtatt | 54 | 0 |
|  | 7 | catggctttcaatgatccaa | aacagcacatgaaacgcaat | 54 | 0 |
|  | 8 | ggcaacatcataccagcaca | ttcaaatgccactctcagga | 54 | 0 |
|  | 9 | cacagatgatgtggcatgttc | tgccttcacacgttggataa | 54 | 0 |
|  | 10 | atcaatggctcatgcactgg | cctgcccccaatctttattt | 54 | 0 |
|  | 11 | taggggatccatcacaggtc | tctctggtgccagtgaatgt | 54 | 0 |
|  | 12 | caagcttaccaatgcttttgc | tgccctttaggtgattcattg | 54 | 0 |
|  | 13 | gcaaaattttccagtctgta | cttgctgtgctgtgctgtct | 54 | 0 |
|  | 14 | ggtctccatccatgagcact | ggcaggcatttttacttgga | 54 | 0 |
|  | 15a | catttgctaactctgaaatgtga | TTGTGCTTTTGGATCTGAACA | 54 | 0 |
|  |  |  |  |  |  |

**Figure A. Next generation sequencing workflow**


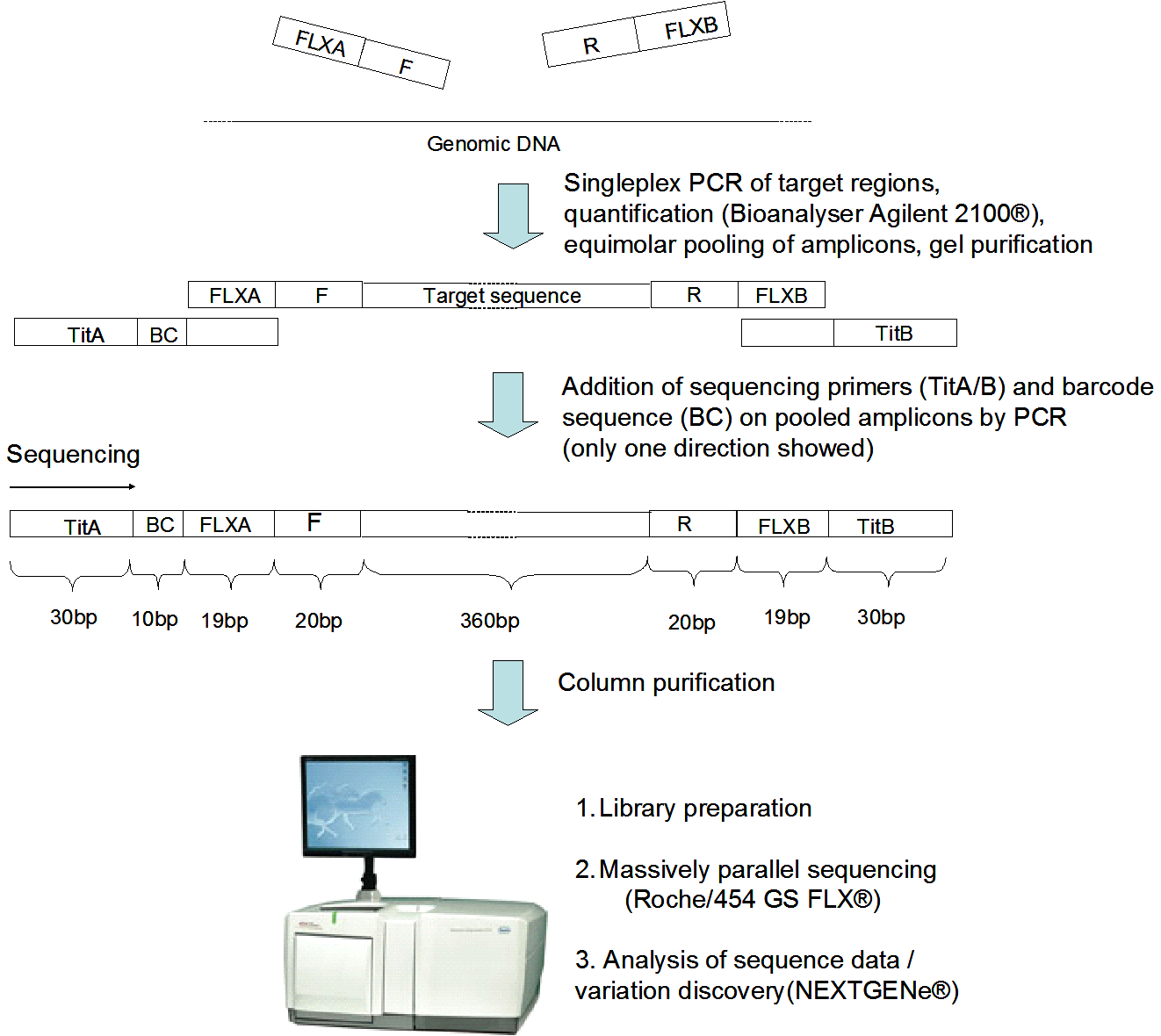


F: forward sequence specific primer (refer to Table A)

R: reverse sequence specific primer (refer to Table A)

FLXA or FLXB: GS FLX primer A or B sequence (Roche/454)

TitA or TitB: GS FLX Titanium sequencing primer (Roche/454)

BC: patient specific barcode sequence
