# Supplementary material for: A founder mutation in the PEX6 gene is responsible for increased incidence of Zellweger syndrome in a French Canadian population
Source: BMC Med Genet. 2012 Aug 15;13:72. doi: 10.1186/1471-2350-13-72 (PMC3483250; doi:10.1186/1471-2350-13-72)
Supplement: Addtional file 2 — Word document, cDNA sequences and predicted proteins of alternate transcripts (Figure B). [file 1471-2350-13-72-S2.doc]

>hg19_refGene_NM_000287 range=chr6:42932073-42946888 5'pad=0 3'pad=0 strand=- repeatMasking=none

ATGGCGCTGGCTGTCTTGCGGGTCCTGGAGCCCTTTCCGACCGAGACACCCCCGTTGGCAGTGCTGCTGCCACCCGGGGGCCCGTGGCCGGCGGCGGAGCTGGGCCTGGTGCTGGCCCTGAGGCCTGCAGGGGAGAGCCCGGCAGGGCCGGCGCTGCTGGTGGCAGCCCTGGAGGGGCCGGACGCGGGCACCGAAGAGCAGGGTCCCGGGCCGCCGCAGCTACTGGTTAGCCGCGCGCTGCTGCGGCTCCTGGCACTGGGCTCCGGGGCCTGGGTGCGGGCGCGGGCGGTGCGGCGGCCCCCGGCGCTAGGTTGGGCACTGCTTGGCACCTCGCTGGGGCCTGGGCTCGGACCGCGAGTCGGGCCGCTGCTGGTGAGGCGCGGAGAGACCCTCCCAGTGCCCGGACCGCGGGTGCTGGAGACGCGGCCGGCGTTGCAAGGGCTGCTGGGCCCAGGGACTCGGCTGGCTGTGACTGAGCTCCGCGGGCGGGCCAGACTGTGTCCAGAGTCTGGGGACAGCAGTCGGCCCCCACCCCCGCCCGTGGTGTCCTCCTTTGCGGTTTCTGGCACAGTGCGGCGACTCCAGGGAGTTCTGGGAGGGACTGGAGATTCACTAGGGGTGAGCCGGAGCTGTCTCCGTGGCCTTGGCCTCTTCCAGGGCGAATGGGTGTGGGTGGCCCAGGCCAGAGAGTCATCGAACACTTCACAGCCGCACTTGGCTAGGGTGCAGGTCCTAGAACCTCGCTGGGACCTCTCTGATAGACTGGGACCCGGCTCTGGACCGCTGGGAGAGCCCCTCGCTGACGGACTGGCGCTTGTCCCTGCCACTTTGGCTTTTAATCTTGGCTGTGACCCCCTGGAAATGGGAGAGCTCAGAATTCAGAGGTACTTGGAAGGCTCCATCGCCCCTGAAGACAAAGGAAGCTGCTCATTGCTGCCTGGGCCTCCATTTGCCAGAGAGTTACACATCGAAATTGTGTCTTCTCCCCACTACAGCACTAATGGAAATTATGACGGTGTTCTTTACCGGCACTTTCAGATACCCAGGGTAGTCCAGGAAGGGGATGTTCTATGTGTGCCAACAATTGGGCAAGTAGAGATCCTGGAAGGAAGTCCAGAGAAACTGCCCAGGTGGCGGGAAATGTTTTTTAAAGTGAAGAAAACAGTTGGGGAAGCTCCAGATGGACCAGCCAGTGCCTACTTGGCCGACACCACCCATACCTCCTTGTACATGGTGGGTTCTACCCTGAGCCCTGTTCCATGGCTCCCTTCAGAGGAATCCACTCTCTGGAGCAGTTTGTCTCCTCCAGGCCTGGAGGCCTTGGTGTCTGAACTCTGTGCTGTCCTGAAGCCTCGCCTCCAGCCAGGGGGTGCCCTGCTGACAGGAACTAGCAGTGTCCTTCTACGGGGCCCCCCAGGCTGTGGGAAGACCACAGTAGTTGCTGCTGCCTGTAGTCACCTTGGGCTCCACTTACTGAAGGTGCCCTGCTCCAGCCTCTGTGCAGAAAGTAGTGGGGCTGTGGAGACAAAACTGCAGGCCATCTTCTCCCGGGCCCGCCGTTGCCGGCCTGCAGTCCTGTTGCTCACAGCTGTGGACCTTCTGGGCCGGGACCGTGATGGGCTGGGTGAGGATGCCCGTGTGATGGCTGTGCTGCGTCACCTCCTCCTCAATGAGGACCCCCTCAACAGCTGCCCTCCCCTCATGGTTGTGGCCACCACAAGCCGGGCCCAGGACCTGCCTGCTGATGTGCAGACAGCATTTCCTCATGAGCTCGAGGTGCCTGCTCTGTCAGAGGGGCAGCGGCTCAGCATCCTGCGGGCCCTCACTGCCCACCTTCCCCTGGGCCAGGAGGTGAACTTGGCACAGCTAGCACGGCGGTGTGCAGGCTTTGTGGTAGGGGATCTCTATGCCCTTCTGACCCACAGCAGCCGGGCAGCCTGCACCAGGATCAAGAACTCAGGTTTGGCAGGTGGCTTGACTGAGGAGGATGAGGGGGAGCTGTGTGCTGCCGGCTTTCCTCTCCTGGCTGAGGACTTTGGGCAGGCACTGGAGCAACTGCAGACAGCTCACTCCCAGGCCGTTGGAGCCCCCAAGATCCCCTCAGTGTCCTGGCATGATGTGGGTGGGCTGCAGGAGGTGAAGAAGGAGATCCTGGAGACCATTCAGCTCCCCCTGGAGCACCCTGAGCTACTGAGCCTGGGCCTGAGACGCTCAGGCCTTCTGCTCCATGGGCCCCCTGGCACCGGCAAGACCCTTCTGGCCAAGGCAGTAGCCACTGAGTGCAGCCTTACCTTCCTCAGCGTGAAGGGGCCAGAGCTCATTAACATGTATGTGGGCCAAAGTGAGGAGAATGTGCGGGAAGTGTTTGCCAGGGCCAGGGCTGCAGCTCCATGCATTATCTTCTTTGATGAACTGGACTCTTTGGCCCCAAGCCGGGGGCGAAGTGGAGATTCTGGAGGAGTGATGGACAGGGTGGTGTCTCAGCTCCTTGCCGAGCTAGATGGGCTGCACAGCACTCAGGATGTGTTTGTGATTGGAGCCACCAACAGACCAGATCTCCTGGACCCTGCCCTTCTGCGGCCTGGCAGATTTGACAAGCTGGTGTTTGTGGGGGCAAATGAGGACCGGGCCTCCCAGCTACGCGTTCTAAGTGCCATCACACGCAAATTCAAGCTAGAGCCATCTGTGAGCCTGGTAAACGTGCTAGATTGCTGCCCTCCCCAGCTGACGGGCGCGGACCTCTACTCTCTCTGCTCTGATGCTATGACAGCTGCCCTCAAACGCAGGGTTCATGACCTGGAGGAAGGGCTGGAGCCAGGTAGCTCAGCACTGATGCTCACCATGGAGGACTTGCTGCAGGCTGCCGCCCGGCTGCAACCCTCAGTCAGTGAGCAGGAGCTGCTCCGGTACAAGCGCATCCAGCGCAAGTTTGCTGCCTGCTAG

Human Mutant 1 cDNA (cryptic site 1; c.226)

ATGGCGCTGGCTGTCTTGCGGGTCCTGGAGCCCTTTCCGACCGAGACACCCCCGTTGGCAGTGCTGCTGCCACCCGGGGGCCCGTGGCCGGCGGCGGAGCTGGGCCTGGTGCTGGCCCTGAGGCCTGCAGGGGAGAGCCCGGCAGGGCCGGCGCTGCTGGTGGCAGCCCTGGAGGGGCCGGACGCGGGCACCGAAGAGCAGGGTCCCGGGCCGCCGCAGCTACTGAGGTACTTGGAAGGCTCCATCGCCCCTGAAGACAAAGGAAGCTGCTCATTGCTGCCTGGGCCTCCATTTGCCAGAGAGTTACACATCGAAATTGTGTCTTCTCCCCACTACAGCACTAATGGAAATTATGACGGTGTTCTTTACCGGCACTTTCAGATACCCAGGGTAGTCCAGGAAGGGGATGTTCTATGTGTGCCAACAATTGGGCAAGTAGAGATCCTGGAAGGAAGTCCAGAGAAACTGCCCAGGTGGCGGGAAATGTTTTTTAAAGTGAAGAAAACAGTTGGGGAAGCTCCAGATGGACCAGCCAGTGCCTACTTGGCCGACACCACCCATACCTCCTTGTACATGGTGGGTTCTACCCTGAGCCCTGTTCCATGGCTCCCTTCAGAGGAATCCACTCTCTGGAGCAGTTTGTCTCCTCCAGGCCTGGAGGCCTTGGTGTCTGAACTCTGTGCTGTCCTGAAGCCTCGCCTCCAGCCAGGGGGTGCCCTGCTGACAGGAACTAGCAGTGTCCTTCTACGGGGCCCCCCAGGCTGTGGGAAGACCACAGTAGTTGCTGCTGCCTGTAGTCACCTTGGGCTCCACTTACTGAAGGTGCCCTGCTCCAGCCTCTGTGCAGAAAGTAGTGGGGCTGTGGAGACAAAACTGCAGGCCATCTTCTCCCGGGCCCGCCGTTGCCGGCCTGCAGTCCTGTTGCTCACAGCTGTGGACCTTCTGGGCCGGGACCGTGATGGGCTGGGTGAGGATGCCCGTGTGATGGCTGTGCTGCGTCACCTCCTCCTCAATGAGGACCCCCTCAACAGCTGCCCTCCCCTCATGGTTGTGGCCACCACAAGCCGGGCCCAGGACCTGCCTGCTGATGTGCAGACAGCATTTCCTCATGAGCTCGAGGTGCCTGCTCTGTCAGAGGGGCAGCGGCTCAGCATCCTGCGGGCCCTCACTGCCCACCTTCCCCTGGGCCAGGAGGTGAACTTGGCACAGCTAGCACGGCGGTGTGCAGGCTTTGTGGTAGGGGATCTCTATGCCCTTCTGACCCACAGCAGCCGGGCAGCCTGCACCAGGATCAAGAACTCAGGTTTGGCAGGTGGCTTGACTGAGGAGGATGAGGGGGAGCTGTGTGCTGCCGGCTTTCCTCTCCTGGCTGAGGACTTTGGGCAGGCACTGGAGCAACTGCAGACAGCTCACTCCCAGGCCGTTGGAGCCCCCAAGATCCCCTCAGTGTCCTGGCATGATGTGGGTGGGCTGCAGGAGGTGAAGAAGGAGATCCTGGAGACCATTCAGCTCCCCCTGGAGCACCCTGAGCTACTGAGCCTGGGCCTGAGACGCTCAGGCCTTCTGCTCCATGGGCCCCCTGGCACCGGCAAGACCCTTCTGGCCAAGGCAGTAGCCACTGAGTGCAGCCTTACCTTCCTCAGCGTGAAGGGGCCAGAGCTCATTAACATGTATGTGGGCCAAAGTGAGGAGAATGTGCGGGAAGTGTTTGCCAGGGCCAGGGCTGCAGCTCCATGCATTATCTTCTTTGATGAACTGGACTCTTTGGCCCCAAGCCGGGGGCGAAGTGGAGATTCTGGAGGAGTGATGGACAGGGTGGTGTCTCAGCTCCTTGCCGAGCTAGATGGGCTGCACAGCACTCAGGATGTGTTTGTGATTGGAGCCACCAACAGACCAGATCTCCTGGACCCTGCCCTTCTGCGGCCTGGCAGATTTGACAAGCTGGTGTTTGTGGGGGCAAATGAGGACCGGGCCTCCCAGCTACGCGTTCTAAGTGCCATCACACGCAAATTCAAGCTAGAGCCATCTGTGAGCCTGGTAAACGTGCTAGATTGCTGCCCTCCCCAGCTGACGGGCGCGGACCTCTACTCTCTCTGCTCTGATGCTATGACAGCTGCCCTCAAACGCAGGGTTCATGACCTGGAGGAAGGGCTGGAGCCAGGTAGCTCAGCACTGATGCTCACCATGGAGGACTTGCTGCAGGCTGCCGCCCGGCTGCAACCCTCAGTCAGTGAGCAGGAGCTGCTCCGGTACAAGCGCATCCAGCGCAAGTTTGCTGCCTGCTAG

Human Mutant 2 cDNA (cryptic site 2; c.619)

ATGGCGCTGGCTGTCTTGCGGGTCCTGGAGCCCTTTCCGACCGAGACACCCCCGTTGGCAGTGCTGCTGCCACCCGGGGGCCCGTGGCCGGCGGCGGAGCTGGGCCTGGTGCTGGCCCTGAGGCCTGCAGGGGAGAGCCCGGCAGGGCCGGCGCTGCTGGTGGCAGCCCTGGAGGGGCCGGACGCGGGCACCGAAGAGCAGGGTCCCGGGCCGCCGCAGCTACTGGTTAGCCGCGCGCTGCTGCGGCTCCTGGCACTGGGCTCCGGGGCCTGGGTGCGGGCGCGGGCGGTGCGGCGGCCCCCGGCGCTAGGTTGGGCACTGCTTGGCACCTCGCTGGGGCCTGGGCTCGGACCGCGAGTCGGGCCGCTGCTGGTGAGGCGCGGAGAGACCCTCCCAGTGCCCGGACCGCGGGTGCTGGAGACGCGGCCGGCGTTGCAAGGGCTGCTGGGCCCAGGGACTCGGCTGGCTGTGACTGAGCTCCGCGGGCGGGCCAGACTGTGTCCAGAGTCTGGGGACAGCAGTCGGCCCCCACCCCCGCCCGTGGTGTCCTCCTTTGCGGTTTCTGGCACAGTGCGGCGACTCCAGGGAGTTCTGGGAGGGACTGGAGATTCACTAGGGAGGTACTTGGAAGGCTCCATCGCCCCTGAAGACAAAGGAAGCTGCTCATTGCTGCCTGGGCCTCCATTTGCCAGAGAGTTACACATCGAAATTGTGTCTTCTCCCCACTACAGCACTAATGGAAATTATGACGGTGTTCTTTACCGGCACTTTCAGATACCCAGGGTAGTCCAGGAAGGGGATGTTCTATGTGTGCCAACAATTGGGCAAGTAGAGATCCTGGAAGGAAGTCCAGAGAAACTGCCCAGGTGGCGGGAAATGTTTTTTAAAGTGAAGAAAACAGTTGGGGAAGCTCCAGATGGACCAGCCAGTGCCTACTTGGCCGACACCACCCATACCTCCTTGTACATGGTGGGTTCTACCCTGAGCCCTGTTCCATGGCTCCCTTCAGAGGAATCCACTCTCTGGAGCAGTTTGTCTCCTCCAGGCCTGGAGGCCTTGGTGTCTGAACTCTGTGCTGTCCTGAAGCCTCGCCTCCAGCCAGGGGGTGCCCTGCTGACAGGAACTAGCAGTGTCCTTCTACGGGGCCCCCCAGGCTGTGGGAAGACCACAGTAGTTGCTGCTGCCTGTAGTCACCTTGGGCTCCACTTACTGAAGGTGCCCTGCTCCAGCCTCTGTGCAGAAAGTAGTGGGGCTGTGGAGACAAAACTGCAGGCCATCTTCTCCCGGGCCCGCCGTTGCCGGCCTGCAGTCCTGTTGCTCACAGCTGTGGACCTTCTGGGCCGGGACCGTGATGGGCTGGGTGAGGATGCCCGTGTGATGGCTGTGCTGCGTCACCTCCTCCTCAATGAGGACCCCCTCAACAGCTGCCCTCCCCTCATGGTTGTGGCCACCACAAGCCGGGCCCAGGACCTGCCTGCTGATGTGCAGACAGCATTTCCTCATGAGCTCGAGGTGCCTGCTCTGTCAGAGGGGCAGCGGCTCAGCATCCTGCGGGCCCTCACTGCCCACCTTCCCCTGGGCCAGGAGGTGAACTTGGCACAGCTAGCACGGCGGTGTGCAGGCTTTGTGGTAGGGGATCTCTATGCCCTTCTGACCCACAGCAGCCGGGCAGCCTGCACCAGGATCAAGAACTCAGGTTTGGCAGGTGGCTTGACTGAGGAGGATGAGGGGGAGCTGTGTGCTGCCGGCTTTCCTCTCCTGGCTGAGGACTTTGGGCAGGCACTGGAGCAACTGCAGACAGCTCACTCCCAGGCCGTTGGAGCCCCCAAGATCCCCTCAGTGTCCTGGCATGATGTGGGTGGGCTGCAGGAGGTGAAGAAGGAGATCCTGGAGACCATTCAGCTCCCCCTGGAGCACCCTGAGCTACTGAGCCTGGGCCTGAGACGCTCAGGCCTTCTGCTCCATGGGCCCCCTGGCACCGGCAAGACCCTTCTGGCCAAGGCAGTAGCCACTGAGTGCAGCCTTACCTTCCTCAGCGTGAAGGGGCCAGAGCTCATTAACATGTATGTGGGCCAAAGTGAGGAGAATGTGCGGGAAGTGTTTGCCAGGGCCAGGGCTGCAGCTCCATGCATTATCTTCTTTGATGAACTGGACTCTTTGGCCCCAAGCCGGGGGCGAAGTGGAGATTCTGGAGGAGTGATGGACAGGGTGGTGTCTCAGCTCCTTGCCGAGCTAGATGGGCTGCACAGCACTCAGGATGTGTTTGTGATTGGAGCCACCAACAGACCAGATCTCCTGGACCCTGCCCTTCTGCGGCCTGGCAGATTTGACAAGCTGGTGTTTGTGGGGGCAAATGAGGACCGGGCCTCCCAGCTACGCGTTCTAAGTGCCATCACACGCAAATTCAAGCTAGAGCCATCTGTGAGCCTGGTAAACGTGCTAGATTGCTGCCCTCCCCAGCTGACGGGCGCGGACCTCTACTCTCTCTGCTCTGATGCTATGACAGCTGCCCTCAAACGCAGGGTTCATGACCTGGAGGAAGGGCTGGAGCCAGGTAGCTCAGCACTGATGCTCACCATGGAGGACTTGCTGCAGGCTGCCGCCCGGCTGCAACCCTCAGTCAGTGAGCAGGAGCTGCTCCGGTACAAGCGCATCCAGCGCAAGTTTGCTGCCTGCTAG

>Human NP_000278.3

Malavlrvlepfptetpplavllppggpwpaaelglvlalrpagespagpallvaalegpdagteeqgpgppqll**v**srallrllalgsgawvraravrrppalgwallgtslgpglgprvgpllvrrgetlpvpgprvletrpalqgllgpgtrlavtelrgrarlcpesgdssrpppppvvssfavsgtvrrlqgvlggtgdslg**v**srsclrglglfqgewvwvaqaressntsqphlarvqvleprwdlsdrlgpgsgplgepladglalvpatlafnlgcdplemgelriqrylegsiapedkgscsllpgppfarelhieivssphystngnydgvlyrhfqiprvvqegdvlcvptigqveilegspeklprwremffkvkktvgeapdgpasayladtthtslymvgstlspvpwlpseestlwsslsppglealvselcavlkprlqpggalltgtssvllrgppgcgkttvvaaacshlglhllkvpcsslcaessgavetklqaifsrarrcrpavllltavdllgrdrdglgedarvmavlrhlllnedplnscpplmvvattsraqdlpadvqtafphelevpalsegqrlsilraltahlplgqevnlaqlarrcagfvvgdlyallthssraactriknsglagglteedegelcaagfpllaedfgqaleqlqtahsqavgapkipsvswhdvgglqevkkeiletiqlplehpellslglrrsglllhgppgtgktllakavatecsltflsvkgpelinmyvgqseenvrevfararaaapciiffdeldslapsrgrsgdsggvmdrvvsqllaeldglhstqdvfvigatnrpdlldpallrpgrfdklvfvganedrasqlrvlsaitrkfklepsvslvnvldccppqltgadlyslcsdamtaalkrrvhdleeglepgssalmltmedllqaaarlqpsvseqellrykriqrkfaac

>Human Mut 1 predicted protein (cryptic site 1; c.226)

MALAVLRVLEPFPTETPPLAVLLPPGGPWPAAELGLVLALRPAGESPAGPALLVAALEGPDAGTEEQGPGPPQLLRYLEGSIAPEDKGSCSLLPGPPFARELHIEIVSSPHYSTNGNYDGVLYRHFQIPRVVQEGDVLCVPTIGQVEILEGSPEKLPRWREMFFKVKKTVGEAPDGPASAYLADTTHTSLYMVGSTLSPVPWLPSEESTLWSSLSPPGLEALVSELCAVLKPRLQPGGALLTGTSSVLLRGPPGCGKTTVVAAACSHLGLHLLKVPCSSLCAESSGAVETKLQAIFSRARRCRPAVLLLTAVDLLGRDRDGLGEDARVMAVLRHLLLNEDPLNSCPPLMVVATTSRAQDLPADVQTAFPHELEVPALSEGQRLSILRALTAHLPLGQEVNLAQLARRCAGFVVGDLYALLTHSSRAACTRIKNSGLAGGLTEEDEGELCAAGFPLLAEDFGQALEQLQTAHSQAVGAPKIPSVSWHDVGGLQEVKKEILETIQLPLEHPELLSLGLRRSGLLLHGPPGTGKTLLAKAVATECSLTFLSVKGPELINMYVGQSEENVREVFARARAAAPCIIFFDELDSLAPSRGRSGDSGGVMDRVVSQLLAELDGLHSTQDVFVIGATNRPDLLDPALLRPGRFDKLVFVGANEDRASQLRVLSAITRKFKLEPSVSLVNVLDCCPPQLTGADLYSLCSDAMTAALKRRVHDLEEGLEPGSSALMLTMEDLLQAAARLQPSVSEQELLRYKRIQRKFAAC

>Human Mut 2 predicted protein (cryptic site 2; c.619)

MALAVLRVLEPFPTETPPLAVLLPPGGPWPAAELGLVLALRPAGESPAGPALLVAALEGPDAGTEEQGPGPPQLLVSRALLRLLALGSGAWVRARAVRRPPALGWALLGTSLGPGLGPRVGPLLVRRGETLPVPGPRVLETRPALQGLLGPGTRLAVTELRGRARLCPESGDSSRPPPPPVVSSFAVSGTVRRLQGVLGGTGDSLGRYLEGSIAPEDKGSCSLLPGPPFARELHIEIVSSPHYSTNGNYDGVLYRHFQIPRVVQEGDVLCVPTIGQVEILEGSPEKLPRWREMFFKVKKTVGEAPDGPASAYLADTTHTSLYMVGSTLSPVPWLPSEESTLWSSLSPPGLEALVSELCAVLKPRLQPGGALLTGTSSVLLRGPPGCGKTTVVAAACSHLGLHLLKVPCSSLCAESSGAVETKLQAIFSRARRCRPAVLLLTAVDLLGRDRDGLGEDARVMAVLRHLLLNEDPLNSCPPLMVVATTSRAQDLPADVQTAFPHELEVPALSEGQRLSILRALTAHLPLGQEVNLAQLARRCAGFVVGDLYALLTHSSRAACTRIKNSGLAGGLTEEDEGELCAAGFPLLAEDFGQALEQLQTAHSQAVGAPKIPSVSWHDVGGLQEVKKEILETIQLPLEHPELLSLGLRRSGLLLHGPPGTGKTLLAKAVATECSLTFLSVKGPELINMYVGQSEENVREVFARARAAAPCIIFFDELDSLAPSRGRSGDSGGVMDRVVSQLLAELDGLHSTQDVFVIGATNRPDLLDPALLRPGRFDKLVFVGANEDRASQLRVLSAITRKFKLEPSVSLVNVLDCCPPQLTGADLYSLCSDAMTAALKRRVHDLEEGLEPGSSALMLTMEDLLQAAARLQPSVSEQELLRYKRIQRKFAAC

**Figure B. cDNA sequences and predicted proteins of alternate transcripts.** Deleted nucleotides (and amino acids) of exon 1 resulting from the use of cryptic donor splice sites are underlined on the wild type cDNA (and wild type protein) sequence. Positions of cryptic donor splice sites (GT) of exon 1 (c.226 and c.619) are highlighted in red and position of exon 2 acceptor splice site (AG) is highlighted in blue. First deleted amino acid of each alternate transcript is in bold. First 3 amino acids of exon 2 are highlighted in yellow.
